# Effect of Gastroesophageal Reflux Disease on Disease Severity and Characteristics of Lung Functional Changes in Patients with Asthma

**DOI:** 10.15171/jcvtr.2014.016

**Published:** 2014-12-30

**Authors:** Akbar Sharifi, Khalil Ansarin

**Affiliations:** Tuberculosis and Lung Disease Research Center, Tabriz University of Medical Sciences,Tabriz, Iran

**Keywords:** Asthma, Gastroesophageal Reflux, Oscillometry, Respiratory Function Tests

## Abstract

***Introduction:*** Almost one third of patients with asthma have symptomatic evidence for coexisting gastroesophageal reflux disease (GERD), which is thought to be aggravating factor in asthma at least in some cases. We investigated the impact of coexisting GERD on asthma severity and parameters of lung function.

***Methods:*** Ninety two asthma patients diagnosed according to ATS criteria were studied. After full history and physical examination, asthma severity was measured in each patient using asthma control test (ACT). GERD symptoms was verified in each patient. Impulse oscillometry (IOS) and lung volume studies (using body-plethysmography and IOS) were performed. The difference between total airway resistance (TAWR) indicated by resistance at 5 Hz and central airway resistance (CAWR) as indicated by resistance at 20 Hz in oscillometry was calculated and considered as representative of resistance at peripheral airways (PAWR). The relationship between the presences of GERD symptoms, ACT score and parameters of lung function were analyzed.

***Results:*** PAWR and TAWR were both significantly higher in asthmatic patients with GERD symptoms than patients without GERD symptoms (256.64±161.21 versus 191.68±98.64; P=0.02, and 102.73±122.39 versus 56.76±71.43; P=0.01, respectively). However, no significant difference was noted in mean values of ACT, FEV1 (forced expiratory volume in 1 sec), FVC (forced vital capacity), PEF (peak expiratory flow), and CAWR in these two groups.

***Conclusion:*** These findings suggest that the severity of asthma as measured by ACT score is not different in patients with and without GERD symptoms. However, total and peripheral airway resistance measured by IOS is significantly higher in asthmatic patients with GERD symptoms.

## Introduction

Asthma and gastroesophageal reflux disease (GERD) are both common, very often coexist, exert a significant effect on the quality of life of patients and specially patients with difficult-to-control asthma.^1-8^ The prevalence of asthma in patients suffering from GERD was greater than the control group.^9,10^ Reflux may precipitate asthma by variety of mechanisms including a vagal reflex activated by mere entrance of gastric contents to esophagus or recurrent micro-aspiration of gastric fluid into the upper airways. Moreover, asthma can precipitate reflux mechanism via widening of the extent of intra-thoracic pressure swings. Furthermore, in patients with asthma the presence of GERD may aggravate bronchoconstriction in response to a variety of stimuli.^11,12^ In agreement with this relationship, studies on medical or surgical treatment of reflux disease were able to alleviate the respiratory symptoms in these patients.^13,14^

The asthma control test (ACT) provided by Global Initiative for Asthma (GINA) has shown to be useful in management of asthma in facilitating the asthma control.^15,16^

Lung function test is useful in evaluating and measuring different aspects of the respiratory system performance. Several different parameters of lung function are believed to reflect the resistance at different levels of central or peripheral airways. Airway resistance measured by either methods of body-plethysmography or impulse oscillometry (IOS) has been proposed as indicator of the site of obstruction in asthma.^17,18^ In addition, some of the parameters of lung function testing have been suggested to reflect distal airway function including closing volume [RV (residual volume), RV/TLC (residual volume over total lung capacity)], forced expiratory flow at 25% to 75% of FVC, FEV1/FVC ratio and low frequency dependence of resistance. On the other hand, some other parameters including FEV1, specific airway conductance (SGW) and high frequency dependence of resistance have been regarded as indicators of proximal airway function.^19^

To date, a few researches have evaluated the relationships between GERD and composite measures of asthma control or physiologic alterations of lung function.^20-22^

Asthma is a syndrome of symptoms and with different patterns of pathological and clinical characteristics originating from potentially different causes of the disease. This study was designed with the aim of assessing any link between GERD as can be inferred by presence of symptoms and in site of (central or peripheral) airway obstruction and severity of asthma.

## Materials and methods

In a cross-sectional study, we recruited 92 adult stable asthmatic patients diagnosed by ATS guidelines who were followed up regularly by a pulmonologist in an outpatient clinic of Tabriz University of Medical Sciences. All patients underwent a detailed clinical history and full physical examination and the presence of at least once weekly GERD symptoms such as heartburn, regurgitation, dysphasia, chest pain, hypersalivation, globus sensation, and odynophagia was verified. The severity of asthma was recorded and graded by ACT score. Considering the ACT score, asthma patients classified in to well-controlled (ACT score ≥20), not well-controlled (ACT score 15-20), and uncontrolled (ACT score <15). All patients were advised to discontinue short acting bronchodilator therapy and exercising for at least eight hours before performing pulmonary function test. The patients first performed IOS measurements (using Master Screen, Jaeger, Germany) and then dynamic airflow and static lung volume measurements were done with body plethysmography (Jaeger, Germany). The body plethysmograph and IOS was calibrated daily to ensure accurate measurements.

Patients were excluded from study if they had a history of current or past smoking more than 10 pack-years, evidence of current airway infection, acute exacerbation within the past one month, any cardio-respiratory disease other than asthma or those who were unable to adequately perform spirometry or impulse oscillometry.

All physiologic parameters including FEV1, FVC, FEF25%, FEF50%, FEF75%, FRC (functional residual capacity), FRC/TLC, RV, RV/TLC, total airway resistance (R total), effective airway resistance measured by body-plethysmography and central airway resistance (CAWR) indicated by resistance at 20 Hz, and resistance at total airways (TAWR) indicated by resistance at 5 Hz measured by IOS are expressed as percentages of their predicted values. The difference between TAWR and CAWR measured in oscillometry was calculated and considered as representative of resistance at peripheral airways (PAWR). Student’s t-test was used to examine the relationshipbetween the presences of GERD symptoms and severity of asthma as indicated by ACT clinical score and changes in lung function parameters. Data analysis were performed using SPSS 15 and P<0.05 was considered statistically significant.

## Results

**Figure 1:**
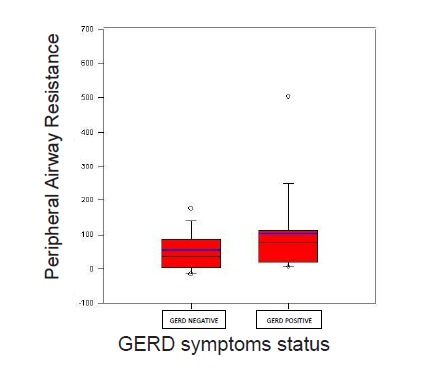
Peripheral airway resistance measured in impulse oscillometry of asthmatic patients with and without GERD symptoms.

**Figure 2:**
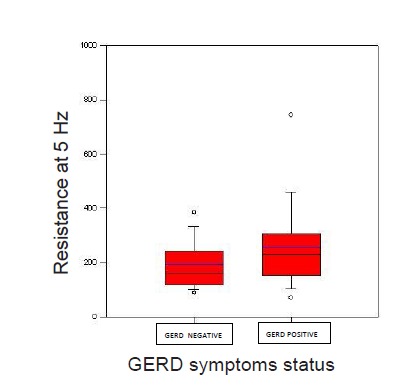
Total airway resistance measured in impulse oscillometry of asthmatic patients with and without GERD symptoms.

Thirty five women and 57 men with asthma (mean age ± SD: 41±14 years) were evaluated in this study. Weekly GERD symptoms were reported in 27.2% of patients. There was no significant difference between subjects with and without GERD symptoms in terms of age, gender, or body mass index (Table 1, P>0.05). The differences in markers of asthma severity including magnitudeof ACT score and lung function parameters in two groups of subjects with and without GERD symptoms are illustrated in Table 1, Figures 1and 2. TAWR and PAWR were both significantly higher in asthmatic patients with GERD symptoms than those without GERD symptoms.

**Table 1:** Differences in parameters of asthma severity in two groups of patients with and without GERD symptoms

**Characteristic**	**With GERD Symptoms**	**Without GERD symptoms**	**P-value**
Age (years)	39.7	41.4	0.57
Sex (numbers F/M)	34/33	15/10	0.29
BMI (Kg/M^2^)	28.7	27.5	0.44
Asthma Control: *(No.)*			
Well controlled (ACT score≥20)	2	7	0.72
Not well controlled (ACT score15-19)	9	23	0.94
Uncontrolled(ACT score< 15)	14	37	0.93
Body Plethysmography (all in % predicted)	Mean ± SD	Mean ± SD	
FEV_1_/FVC	66.74 ±16.69	69.19±12.05	0.43
FEV_1_	69.69±22.86	76.39±24.67	0.24
ITGV/TLC	103.85±19.01	99.83 ±25.05	0.47
RV/TLC	120.64±47.92	116.51±39.74	0.67
FEF50%	44.96±29.64	50.40±31.03	0.43
FEF25-75%	43.06±46.63	48.98±34.45	0.47
Raw Total	236.95±124.02	201.30±122.51	0.26
Raw Effective	216.65±115.91	176.14±107.22	0.16
Impulse Oscillometry: (KPa L^-1^S^-1^)	Mean ± SD	Mean ± SD	
Resistance at 5Hz(TAWR)	256.64±161.21	191.68±98.64	0.02
Resistance at 20Hz	153.90±55.96	137.33±46.44	0.15
PAWR	102.73±122.39	56.76±71.43	0.02

GERD, Gastro-esophageal reflux; IOS, Impulse oscillometry; TAWR, Total airway resistance (R_5_ Hz); PAWR, Peripheral airways resistance (R_5_-R_20_); ACT, Asthma control test; FEV1, Forced expiratory volume in 1 second; FVC, Forced vital capacity; FEF 25% to 75%, Forced expiratory flow at of 25% to 75% FVC; FEF 50%, Forced expiratory flow at the beginning point of 50% of vital capacity; RV, Residual volume; TLC, Total lung capacity; ITGV, Intrathoracic gas volume (functional residual capacity); SGW, Specific airway conductance; Raw Total, Total airway resistance; Raw Effective, effective airway resistance; BMI, Body mass index.

No significant differences were noted in mean values of ACT, FEV1, FVC, PEF, and CAWR between the two studied groups (P>0.05). Analysis of data investigating relationship between presence of GERD symptoms and asthma severity categories based on their ACT score did not reveal any significant association. Furthermore, an analysis with creating new group of patients with summation of well controlled and non-well controlled patients with asthma (ACT≥ 15) in comparison to uncontrolled patients (ACT<15) did not reveal any association to presence of GERD symptoms.

## Discussion

Analysis of data revealed an interesting finding of a significantly higher peripheral airway resistance measured by IOS in asthmatic patients with GERD symptoms compared to asthmatic subjects without these symptoms while no such a difference was detected in indices of central airway resistance between these two groups. In addition, no significant association was noted between presence and absence of reflux symptoms and spirometric values, total airway resistance, and lung volume parameters measured by body-plethysmography. Previous epidemiologic and interventional studies reported connection between GERD and asthma. According to these studies, asthmatic patient with symptoms of GERD had also more pulmonary complaints than those without reflux. Many studies have suggested that treatment of GERD can lead to symptomatic improvement of asthma, less drug use in asthma and improvement in pulmonary function tests. In addition, according to some studies, early diagnosis and treatment of GERD often improves asthma control status.^13,14,23^

Asthma is a multifaceted disease and therefore several systems have been used to measure its severity or activity. These systems are based on parameters of lung function (spirometry and airway resistance) and/or clinical assessment tools (measuring symptoms and quality of life). However, each of these systems tended to measure and reflect a particular aspect of asthma and at least partially independent of the other systems. The ACT is a symptom-based questionnaire system designed to evaluate asthma control status. Some studies have compared the validity of ACT as a measure of asthma control GINA classification status and FEV1 value of the individuals.^16^ Also the Persian version of ACT was developed and tested to be valid and reliable for assessing the status of asthma control.^23-25^

Among GERD symptoms, heartburn is the most predominant symptom in general population and has been reported as a typical symptom of GERD and reasonably specific for diagnosis of this condition.^26^ According to a review of studies from many different locations, the prevalence of GERD symptoms in patients with asthma was 59.2%^1^, while this prevalence was 27.2% in our study. Several factors may underlie the lower prevalence of these symptoms in patients with asthma in our study compared to other reports. Among these factors a plausible explanation may be higher prevalence of *H. pylori* infection in some parts of the world and the different potency between its strains in protective effects against development of GERD.^9,27-30^

Inflammation is thought to be the main factor implicated in asthma pathophysiology involving both central and peripheral airways. However, the differential involvement of central versus peripheral airways in asthma may be a factor in influencing different presentation and pathways of disease progression. Although inflammatory process appears to be similar in central and peripheral airways, relative magnitude of inflammation seems to be more important in peripheral airways in patients with severe asthma.^31,32^ The IOS provides assessments of mechanics of central and peripheral airways.^33^ Previous studies demonstrated that the resistance measured by IOS hadequal or in some study higher sensitivity than FEV1 measured by spirometry for detecting airway obstruction and assessing the efficacy of asthma therapy.^34-36^ Additionally, studies have revealed that airway resistance measured by IOS is able to detect airway hyper-responsiveness, to measure airflow obstruction and to assess bronchodilator response in patients with asthma.^33^In addition, airway resistance measured by IOS correlates better with methacholine-induced symptoms and dyspnea scores in asthmatic patients compared to parameters of spirometry.^37^ Many studies have suggested PAWR and R5 Hz measured with IOS as indicative of small airway resistance.^38^ In the present study, variations of these parameters of peripheral airway resistance were closely related to GERD symptoms, while no relationship was detected between symptoms of GERD and R20 Hz, FEV1, nor the resistance or lung volume changes assessed by body-plethysmography.^36,38^ These findings suggest that R5 Hz seems to be influenced more significantly than FEV1 and R20 Hz by chronic backflow of gastric contents into the esophagus.^36,39-41^

We found significant difference in distal airway physiologic functional values, measured by IOS in asthmatic patients with GERD symptoms compared to patients without GERD symptoms. Resistance at 5 Hz was significantly higher in asthmatic patients with GERD symptoms. This reveals a possible concealed relationship between GERD and more dominant peripheral airway involvement in patients with asthma with no significant difference in clinical severity of the disease. These results are in line with the concept that asthma triggered by GERD has more distal airway involvement compared to other asthmatics. GERD may initiate or aggravate asthma via several different mechanisms including vagal reflex triggered by low pH or merely by bulk effect of gastric fluid influx into the esophagus, or possibly by micro-aspiration of the refluxed fluid into the trachea.^42,43^

In experimental studies, exposure of the trachea to acid increases airway resistance, which can be inhibited by previous surgical or pharmacological blockade of the vagus nerve.^44,45^

One possible explanation of more dominant peripheral airway obstruction in patients with GERD may be lying on the greater influence of reflex bronchospasm secondary to acid exposure of distal esophagus than the direct effect of micro-aspiration on proximal airways.^46^ Of note was the detection of this relationship with peripheral parameters of small airway involvement by IOS, while no difference in parameters of spirometry or lung volume study, which may lie on the difference in delicacy in detection between these techniques which is beyond the scope of this study.^36,42^

This theory is also in line with the theory that there is no need for proximal esophageal acid exposure and actual aspiration of the acid to trachea for development of bronchospasm.^12^ These findings, if proved by further studies using probes for direct detection of reflux, may infer that measures to reduce gastroesophageal reflux may have significant effect on the magnitude and characteristics of airway involvement in patients with asthma. Our findings also suggest that objective monitoring of peripheral airway resistance measured by IOS may be a better parameter to follow up asthmatic patientswith symptoms of GERD and to monitor the effects of therapeutic interventions.

## Ethical issues

The study was approval by the local ethics committee.

## Competing interests

Authors declare no conflict of interests in this study.
